# Effects of high-sensitivity C-reactive protein and left ventricular hypertrophy on cognitive function in hemodialysis patients

**DOI:** 10.1080/0886022X.2025.2450522

**Published:** 2025-01-16

**Authors:** Yu Zhang, Yu-lu Gu, Wan-fen Zhang, Xiao-ping Li, Lin-fang Xu, Tong-qiang Liu

**Affiliations:** Department of Nephrology, the Affiliated Changzhou Second People’s Hospital with Nanjing Medical University, Changzhou, China

**Keywords:** Hemodialysis, cognitive impairment, high-sensitivity C-reactive protein, left ventricular hypertrophy, synergy

## Abstract

**Objectives:**

To examine the effects of high-sensitivity C-reactive protein (hs-CRP) and left ventricular hypertrophy (LVH) on the cognitive function of hemodialysis (HD) patients, and to explore the relationship between hs-CRP, LVH, and cognitive impairment (CI).

**Methods:**

A cross-sectional study was conducted on 232 HD patients. Besides, general clinical data were gathered, and patients’ cognitive functions were assessed using the Beijing version of the Montreal Cognitive Assessment (MoCA-BJ). CI risk factors were screened using logistic regression modeling based on hs-CRP values (low risk <1 mg/L, intermediate risk 1–3 mg/L, and high risk >3 mg/L) and LVH status (normal and hypertrophic) groupings. The synergistic effect of hs-CRP and LVH on CI was also analyzed using the EpiR package.

**Results:**

Among HD patients, 122 (52.59%) patients had CI. Multifactorial logistic regression analysis showed that the following factors were associated with an increased risk of CI in HD patients: age (OR = 1.048; 95% CI 1.014–1.083; *p* = 0.005), LVH (OR = 3.741; 95% CI 1.828–7.657; *p* < 0.001), and high-risk hs-CRP levels (>3 mg/L; OR = 3.238; 95% CI 1.349–7.768; *p* = 0.009). In addition, there was a significant synergy between hs-CRP high risk (>3 mg/L) and LVH.

**Conclusion:**

Age, LVH, and high risk of hs-CRP (>3 mg/L) were independent risk factors for CI in HD patients. Moreover, HD patients with both hs-CRP high risk (>3.0 mg/L) and LVH were at higher risk of developing CI, and lowering hs-CRP levels and preventing LVH may prevent CI.

## Graphical Abstract

**Figure:**
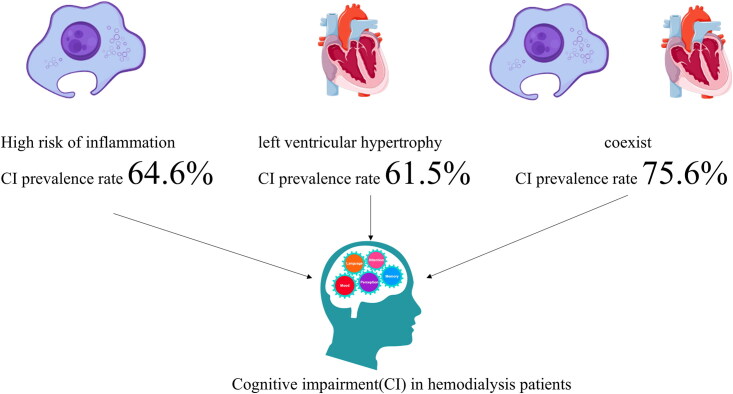

## Introduction

Hemodialysis (HD) is the most widely available option for sustaining life in patients with end-stage renal disease (ESRD). There are approximately 3 million HD patients worldwide and this number is expected to reach 5.4 million by 2030 [1]. Cognitive impairment (CI) has been reported to be prevalent in 87% of HD patients worldwide [2,3]. CI is an intermediate clinical state between cognitive aging and dementia, and in many cases, it precedes and leads to dementia [4]. Notably, the mortality rate of HD patients with comorbid CI is 1.7 to 2.5 times higher than that of patients without CI [5]. CI seriously affects patients’ ability to care for themselves and adhere to treatments, thereby reducing their survival and quality of life, and increasing the burden on their families and society. Therefore, it is necessary to identify CI risk factors early so that appropriate interventions can be implemented.

Low-grade chronic inflammation is prevalent in HD patients [6], which is a mild, slow, persistent pathologic damage resulting from a combination of factors including toxins, complement, and immune complexes in the body [7]. This inflammatory state can further cause malnutrition [8], cardiovascular disease [9], atherosclerosis [10], and cognitive impairment [11]. High-sensitivity C-reactive protein (hs-CRP) is a sensitive marker of systemic low-grade inflammation [12], and its predictive value for CI has been confirmed [9,11]. However, the complex physiopathologic changes in HD patients cause fluctuations in hs-CRP levels, making it inadequate to rely solely on hs-CRP for accurately detecting CI.

Left ventricular hypertrophy (LVH) is an early cardiac structural alteration associated with cardiovascular risk factors [13] and serves as a significant predictor of cardiovascular morbidity and mortality [14–17], and 49–74% of HD patients exhibit LVH on echocardiography [18]. As the heart is responsible for driving cerebral perfusion, chronic reductions in cardiac output may compromise the functional integrity of the brain [19–21], potentially leading to CI [16,22]. However, each hemodialysis procedure causes significant changes in the patient’s internal environment. A relatively stable cardiac structure can provide useful predictive information to reduce the impact of hs-CRP fluctuations [11,23]. Consequently, we anticipate that hs-CRP and LVH can enhance the precision of CI identification and allow exploration of their interaction (synergy).

## Methods

### Patients and study design

**Figure 1.**
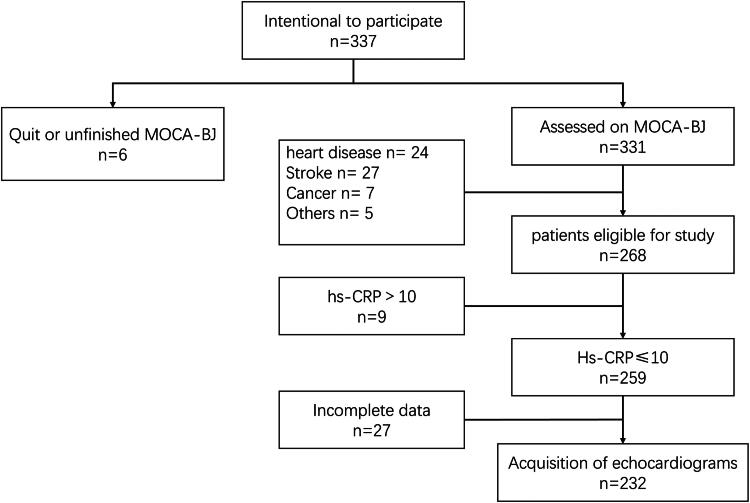
Flow chart.

A retrospective collection of 232 patients treated with HD at our institution from July 2019 to January 2024 was examined (Figure 1). Inclusion criteria were as follows: age between 18 and 80 years; receiving HD therapy for ≥3 months and performing HD three times a week; and being able to undergo the questionnaire assessment. Exclusion criteria were as follows: those with coronary artery disease, hypertrophic cardiomyopathy, dilated cardiomyopathy, severe heart valve disease, congenital heart disease, severe arrhythmias, and other cardiac diseases that impact the structure and function of the heart; heart failure (CHF) (New York Heart Association [NYHA] class II or higher); history of cerebrovascular accidents including cerebral infarcts and/or hemorrhages; and history of major trauma in the month before the collection, history of mental illness, namely, depression or schizophrenia; acute infectious states; and other serious health conditions, including malignancy; and history of renal transplantation. The study received approval from the Ethics Committee of Changzhou Second People’s Hospital ([2024]KY129-01).

#### Data collection

Demographic, clinical, and laboratory data were retrieved from medical records. Demographic and clinical characteristics comprised age, sex, height, weight, literacy (categorized by the highest level of education attained: elementary school or below, middle school, high school, or higher), duration of hemodialysis, smoking status, pre-hemodialysis blood pressure levels, prevalence of diabetes mellitus and hypertension, use of hypertension medications, and lipid-lowering medications. Blood collection was performed before the patient was fasted and short gap dialysis was started. Laboratory data included hemoglobin, high-sensitivity C-reactive protein, glycosylated hemoglobin, fasting glucose, blood urea nitrogen, blood creatinine, blood uric acid, serum albumin, total cholesterol, high-density lipoprotein cholesterol, low-density lipoprotein cholesterol, triglycerides, parathyroid hormone, blood potassium, blood sodium, blood calcium, and inorganic phosphorus. Dry weight was defined as achieving an edema-free state without upright hypotension at the end of the HD session. Interdialytic weight gain (IDWG) was calculated as the pre-dialysis weight before HD treatment minus the post-dialysis weight from the previous dialysis session. IDWG% = IDWG (kg)/dry weight (kg) * 100 [24]. Hyponatremia was defined as SNa ≤ 135 mmol/L using the following formula for correcting the sNa concentration based on serum glucose concentration: Measured Sodium Concentration + [0.016 × (18 × Serum Glucose (mmol/L) − 100)] [25]. Intradialytic hypotension (IDH) is defined as a decrease in systolic blood pressure (pre-dialysis SBP – lowest SBP on dialysis) ≥30 mmHg [26]. Metabolic acidosis (MA), which is defined as a serum bicarbonate level <22 mmol/L [27]. In the patient’s dialysis program, hemodialysis and hemodiafiltration (HDF) alternated, which we defined in the article as HDF ≤1 time/week and HDF = 2 times/week.

#### hs-CRP stratification

Elevated serum hs-CRP levels exceeding 10 mg/L were indicative of acute infection, prompting us to exclude this patient cohort from our analysis [28,29]. Based on the Centers for Disease Control and Prevention/American Heart Association (CDC/AHA) guidelines, hs-CRP was categorized into low-risk (<1.0 mg/L), intermediate-risk (1.0–3.0 mg/L), and high-risk (>3.0 mg/L) groups [23].

#### Echocardiography

Echocardiography was performed before the patient was fasted and short gap dialysis was started. Echocardiography was conducted by two trained sonographers using a Philips iE33 Doppler echocardiography system equipped with a real-time 3D probe X3-1 (frequency 1–3 MHz) (Philips, Best, the Netherlands). Measurements included left ventricular end-diastolic internal diameter (LVDd), interventricular septal thickness (IVST), left ventricular posterior wall thickness (LVPWT), and ejection fraction (EF). Performed according to the American Society of Echocardiography/European Society of Cardiovascular Imaging quantification document [30], LV mass: LVM (g) = 0.8 × 1.04 × [(interventricular septum + left ventricular septal diameter + posterior wall thickness)^3^ − LVPWT^3^] + 0.6 g. Furthermore, LV mass was considered an unadjusted variable normalized by body surface area (BSA) and expressed as the left ventricular mass index (LVMI). The formula for calculating body surface area (BSA) is as follows: BSA (m^2^) = ((height (cm) * weight (kg))/3600)^1/2^. Left ventricular hypertrophy (LVH) was defined as a left ventricular mass index (LVMI) greater than 95 g/m^2^ in women and greater than 115 g/m^2^ in men. Cardiac diastolic function was assessed by a cardiac sonographer and categorized as normal or abnormal.

#### Assessment of cognitive function

Patients were tested for cognitive functioning using the MoCA-BJ, a scale that assesses seven cognitive domains, namely, visuospatial/executive functioning, naming, attention, language, abstraction, delayed recall, as well as orientation. MoCA-BJ scores range from 0 to 30, with a score of <26 suggestive of CI. To adjust for the effects of education, one point was added for patients with an educational level of 12 years or less. A cognitive function assessment was performed in a separate room with 1 medical staff member testing 1 patient [31].

#### Hemodialysis

All patients were dialyzed by WeGo dialyzer, and the ultrafiltration volume and target weight were set according to KDOQI ‘clinical practice guidelines for hemodialysis adequacy’. Patients were treated with bicarbonate buffer for dialysis, Baxter Polyflux14L hollow fiber dialyzer for hemodialysis, and Baxter Revaclear400 hemodiafiltration filter for hemodiafiltration. The blood flow was maintained at 200–280 mL/min, and the dialysate flow was 500 mL/min. Low molecular weight heparin was used for anticoagulation. All patients were treated with arteriovenous fistula or long-term catheter as vascular access.

### Statistical analysis

Data were analyzed by utilizing SPSS version 25.0 (IBM). Normally distributed continuous variables are expressed as the mean and standard deviation. Non-normally distributed continuous variables are expressed as the median and interquartile range. Categorical variables were expressed as frequencies and percentages. To compare the two groups of normal variables, an independent samples t-test was adopted while for skewed and categorical variables, the Mann-Whitney U-test, chi-square test, and Fisher’s exact test were employed. One-way ANOVA and Kruskal–Walli’s test were adopted to analyze multiple groups of variables. Pearson correlation or Spearman rank correlation analysis was used to examine the relationships between variables. Univariate logistic regression analyses were conducted, followed by multivariate regression analyses, with covariates showing a p-value < 0.1 in the univariate regression being selected to identify independent risk factors for CI.

Synergistic effects were analyzed by the EpiR package. Rothman proposed that the evaluation for biological interactions should be on the basis of the additive scale, and three indicators were constructed for the logistic model to evaluate whether there were additive interactions between factors (relative excess risk due to interaction (RERI), attributable proportion (AP), synergy index (SI)). Due to the small number of individuals in the low-risk group, hs-CRP was reclassified for the interaction analysis into two categories: high-risk (>3.0 mg/L) and non-high-risk (≤3.0 mg/L). We assessed synergistic effects using the Rothman synergy index with hs-CRP non-high risk and no LVH as the reference to determine whether the combined effect of hs-CRP and LVH on CI exceeded the sum of the effects of each factor in age-, sex-, and education-adjusted models [32]. The following four groups were compared: no LVH and hs-CRP ≤ 3 mg/L (reference), no LVH and hs-CRP >3 mg/L, LVH and hs-CRP ≤ 3 mg/L, and LVH and hs-CRP >3 mg/L. If the confidence interval of RERI or AP includes 0, or the confidence interval of SI includes 1, it indicates no interaction between these two factors. When SI >1, it is deemed that there is a positive additive interaction between the two factors, that is, synergy. Meanwhile, Bilateral *p* < 0.05 was considered significant.

## Results

### Baseline demographic characteristics and laboratory data

In this cross-sectional study, a total of 232 patients were evaluated by MOCA-BJ. The average age of the patients was 55.50 ± 13.19 years, and the mean BMI was 22.59 ± 3.67 kg/m^2^. Among the patients, 131 (56.5%) were male, and 23 (9.9%) had attained university-level education or higher. Moreover, the median duration of dialysis was 36 months (12 months to 60.75), and the mean values of LVMI and hs-CRP were 129.61 ± 34.42 g/m^2^ and 3.84 ± 2.76 mg/l, respectively. Additionally, in the study population, 94.83% suffered from hypertension, and 39.22% from diabetes. According to the American Society of Echocardiography document, LVH was present in 156 patients (67.24%).

### Clinical characteristics

In the present study, 45 patients (19.4%), 71 patients (30.6%), and 116 patients (50.0%) belonged to the low, intermediate, and high-risk groups, respectively, according to the grouping of hs-CRP levels. As illustrated in Table 1, only the prevalence of CI was significantly different. Further analysis revealed significant differences in the prevalence of CI between the high and low-risk hs-CRP groups (*p* = 0.002) and between the high and intermediate-risk groups (*p* = 0.003). The remaining variables, including age and dialysis duration, were not substantially different, even though they tended to increase with the level of inflammatory markers.

**Table 1.** Comparison of clinical and cardiovascular characteristics based on hs-CRP grouping.

	Low risk:<1 (*n* = 45)	Intermediate risk: 1–3 (*n* = 71)	High risk:>3 (*n* = 116)	*P*
Age (years)	54 (46.5–60)	54 (44–61)	59 (48–69.75)	0.08
Males, n (%)	24 (53.3)	41 (57.8)	66 (56.9)	0.889
BMI (kg/m^2^)	22.48 (21.17–24.99)	22.09 (19.72–24.82)	22.12 (20.31–24.57)	0.856
Smoking, n (%)	17 (37.8)	34 (47.9)	52 (44.8)	0.561
TC (mmol/L)	3.90 (3.32–4.37)	3.84 (3.12–4.41)	3.66 (3.20–4.57)	0.922
Triglyceride (mmol/L)	1.41 (1.04–2.02)	1.76 (1.29–2.17)	1.63 (1.25–2.16)	0.106
HDL-C (mmol/L)	1.02 (0.76–1.29)	0.85 (0.70–1.07)	0.89 (0.75–1.12)	0.089
LDL-C (mmol/L)	1.82 (1.43–2.36)	1.93 (1.44–2.42)	1.82 (1.42–2.20)	0.616
Duration of dialysis (months)	24 (10–38.5)	28 (12–60)	36 (12–72)	0.063
Lipid‐lowering medication, n (%)	23 (51.1)	30 (42.3)	58 (50.0)	0.523
LVH, n (%)	34 (75.6)	40 (56.3)	82 (70.7)	0.053
CI, n (%)	17 (37.8)	30 (42.3)	75 (64.6)	0.001

Values for categorical variables are given as number (percentage); values for continuous variables, as mean ± standard deviation or median [interquartile range].

BMI, Body mass index; TC, Total Cholesterol; HDL-C, High-density lipoprotein cholesterol; LDL-C, Low-density lipoprotein cholesterol; LVH, Left ventricular hypertrophy; CI, Cognitive Impairment.

As demonstrated in Table 2, the prevalence of hypertension, systolic blood pressure (SBP), and diastolic blood pressure (DBP) were higher in the group with LVH in comparison to patients without LVH HD. Regarding hypertension medication, patients with LVH more frequently used β-blockers and calcium channel blockers. The prevalence of CI was higher and the ejection fraction was lower in the LVH group. However, there was no observed difference in diastolic dysfunction, likely because the data included were dichotomous variables based primarily on the judgment of cardiac ultrasonographers. Through correlation analysis, we discovered a statistically significant correlation between LVMI and both hs-CRP (*R* = 0.134, *p* = 0.042) and SBP (*R* = 0.21, *p* = 0.001). But the correlation between LVMI and diastolic blood pressure (*R* = 0.124, *p* = 0.059) was not significant.

**Table 2.** Comparison of clinical and cardiovascular characteristics based on LVH subgroups.

	LVH (−) (*n* = 76)	LVH (+) (*n* = 156)	*p*
Age (years)	54.50 (45.00–68.75)	56.5 (47.00–64.00)	0.897
Males, n (%)	49 (64.5)	82 (52.6)	0.086
BMI (kg/m^2^)	22.42 (20.80–24.81)	22.08 (20.09–24.86)	0.593
Smoking history (%)	37 (48.7)	66 (42.3)	0.359
Hypertension, n (%)	68 (89.5)	152 (97.4)	0.022
DM, n (%)	29 (38.2)	62 (39.7)	0.816
SBP (mmHg)	141.30 ± 19.55	154.37 ± 21.72	<0.001
DBP (mmHg)	78.76 ± 11.14	83.83 ± 12.87	0.004
HR (beats/min)	85.74 ± 11.25	83.26 ± 13.53	0.168
LVEF (%)	60 (56.25–62.00)	57 (54–62)	0.007
Diastolic dysfunction, n (%)	62 (81.6)	131 (84.0)	0.647
Beta-blockers, n (%)	27 (35.5)	79 (50.6)	0.03
CCB, n (%)	35 (46.1)	110 (70.5)	<0.001
ACEI/ARB inhibitors, n (%)	16 (21.1)	29 (18.6)	0.656
hs-CRP (mg/L)	2.74 (1.73–5.58)	3.28 (1.22–6.10)	0.561
CI, n (%)	26 (34.20)	96 (61.5)	<0.001
Duration of dialysis (months)	26 (12–60)	36 (12–72)	0.211
spKt/V	1.33 (1.26–1.47)	1.30 (1.23–1.40)	0.122

Values for categorical variables are given as number (percentage); values for continuous variables, as mean ± standard deviation or median [interquartile range].

LVH, Left ventricular hypertrophy; BMI, Body mass index; DM, Diabetes mellitus; SBP, Systolic Blood pressure; DBP, Diastolic Blood pressure; HR, heart rate; LVEF, left ventricle ejection fraction; CCB, Calcium channel blockers; ACEI, Angiotensin Converting Enzyme Inhibitor; ARB, Angiotensin Receptor Blocker; hs-CRP, high-sensitivity C-reactive protein; CI, Cognitive Impairment.

As shown in Table 3, out of the 232 patients included, 122 (52.59%) patients had a MOCA-BJ score <26, suggesting CI. Patients with CI had a higher mean age, lower education levels, and a longer duration of dialysis. They were more likely to exhibit lower diastolic blood pressure. In laboratory findings, patients with CI were more likely to have lower levels of hemoglobin (HB), albumin, and potassium, as well as higher levels of hs-CRP. In terms of cardiac ultrasound, patients with CI had higher rates of LVMI, LVH ratio, and diastolic dysfunction. In addition, MOCA-BJ score was negatively correlated with hs-CRP (R = −0.193, *p* = 0.003) and LVMI (R = −0.171, *p* = 0.009).

**Table 3.** Clinical characteristics and echocardiographic parameters between CI and non-CI groups.

Variables	Lab normal range	CI (−) (*n* = 110)	CI (+) (*n* = 122)	p
Age (years)		52 (41.75–60)	59 (50.75–71.25)	<0.001
Males, n (%)		63 (57.3)	68 (55.7)	0.814
BMI (kg/m^2^)		22.24 (19.88–24.80)	22.0 (20.62–24.85)	0.779
Smoking history (%)		48 (43.6)	55 (45.1)	0.825
Education (%)				<0.001
≤Elementary school		17 (15.5)	51 (41.8)	
Middle school		52 (47.3)	45 (36.9)	
High school		26 (23.6)	18 (14.8)	
>High school		15 (13.6)	8 (6.6)	
Hypertension, n (%)		105 (95.5)	115 (94.3)	0.682
DM, n (%)		43 (39.1)	48 (39.3)	0.969
SBP (mmHg)		150.25 ± 22.79	149.93 ± 21.10	0.912
DBP (mmHg)		84.26 ± 11.65	80.28 ± 13.04	0.015
Dialysis duration (months)		24 (12–60)	36 (12–72)	0.007
spKt/V		1.31 (1.24–1.46)	1.32 (1.25–1.40)	0.493
LVMI (g/m^2^)		114.97 (94.94–143.63)	132.74 (111.47–159.21)	0.001
LVH, n (%)		60 (54.5)	96 (78.7)	<0.001
LVEF (%)		59.00 (55.00–62.00)	58.00 (54.75–61.00)	0.429
Diastolic dysfunction, n (%)		85 (77.3)	108 (88.5)	0.022
IDWG%		2.89 (1.84–4.00)	2.83 (1.94–3.95)	0.849
UF (L)		1.75 (1.30–2.43)	1.95 (1.50–2.60)	0.127
IDH, n (%)		39 (35.5)	46 (37.7)	0.722
Hyponatremia, n (%)		13 (11.8)	18 (14.8)	0.512
MA, n (%)		22 (20.0)	32 (26.2)	0.262
HDF ≤ 1 (times/week)		78 (70.9)	92 (75.4)	0.439
AVF, n (%)		94 (85.5)	103 (84.4)	0.827
Hemoglobin (g/L)	Male :130 − 175/Female: 115–150	109 (94.00–122.25)	100 (84.00–114.00)	0.012
Hs-CRP (mg/L)	0–10	2.5 (0.96–4.25)	4.89 (1.97–6.54)	<0.001
Glycated hemoglobin (%)	4.0–6.0	5.50 (5.20–6.40)	5.65(5.20–6.40)	0.524
Albumin (g/L)	40–55	40.52 ± 5.48	38.73 ± 5.38	0.013
Glucose (mmol/L)	3.9–6.1	5.70 (4.74–8.25)	5.16 (4.47–7.56)	0.121
BUN (mmol/L)	3.6–9.5	19.37 ± 7.98	18.78 ± 7.51	0.567
Creatinine (µmol/L)	57–111	749.65 (569.25 − 932.33)	727.90 (571.75 − 893.50)	0.509
UA (µmol/L)	208–428	327.87 ± 110.14	326.69 ± 98.37	0.932
TC (mmol/L)	<5.18	3.86 (3.12–4.41)	3.74 (3.26–4.63)	0.998
Triglyceride (mmol/L)	<1.7	1.69 (1.18–2.41)	1.60 (1.18–2.09)	0.342
HDL-C (mmol/L)	1.04–1.55	0.89 (0.73–1.09)	0.92 (0.75–1.13)	0.742
LDL-C (mmol/L)	<3.37	1.82 (1.4–2.21)	1.86 (1.44–2.36)	0.526
PTH (ng/L)	15–65	174.90 (68.75 − 357.43)	182.00 (78.40 − 391.88)	0.482
Potassium (mmol/L)	3.5–5.3	4.54 (4.17–5.10)	4.47 (3.92–4.75)	0.021
Sodium (mmol/L)	137–147	138.65 ± 3.26	138.25 ± 3.87	0.398
Calcium (mmol/L)	2.1–2.55	2.26 ± 0.21	2.23 ± 0.21	0.316
Phosphorus (mmol/L)	0.85–1.5	1.74 (1.43–2.13)	1.69 (1.35–2.04)	0.373

Values for categorical variables are given as number (percentage); values for continuous variables, as mean ± standard deviation or median [interquartile range].

BMI, Body mass index; DM, Diabetes mellitus; SBP, Systolic Blood pressure; DBP, Diastolic Blood pressure; LVMI, Left ventricular Mass Index; LVH, Left ventricular hypertrophy; LVEF, left ventricle ejection fraction; IDWG%: Interdialytic Weight Gain/Dry Weight = (current pre-dialysis weight (kg) – previous post-dialysis weight (kg))/target dry weight (kg) × 100; UF, ultrafiltration volume; IDH, Intradialytic hypotension; MA, metabolic acidosis; HDF, hemodiafiltration; AVF, Arteriovenous fistulas; BUN, blood urea nitrogen; UA, uric acid; TC, Total Cholesterol; HDL-C, High-density lipoprotein cholesterol; LDL-C, Low-density lipoprotein cholesterol; PTH, parathyroid hormone.

### Logistic regression analysis of CI-related factors

As indicated in Table 4, a one-way logistic regression with CI as the dependent variable was first performed, which revealed that age, education, LVH, hs-CRP classification, albumin, potassium, hemoglobin, diastolic blood pressure, and diastolic dysfunction were substantial, and based on the criterion of *p* < 0.1, we expanded the inclusion of dialysis duration, ACEI/ARB, heart rate, and basic human characteristics including gender, BMI for multifactorial regression analysis, which indicated that age (OR = 1.048; 95%CI 1.014–1.083; *p* = 0.005), LVH (OR = 3.741; 95%CI 1.828–7.657; *p* < 0.001), hs-CRP high risk (OR = 3.238; 95%CI 1.349–7.768; *p* = 0.009) was an independent risk factor for CI(Full version in Table S1).

**Table 4.** Logistic regression with CI as dependent variable.

	Univariate analysis		Multivariate analysis	
	OR (95% CI)	P	OR (95% CI)	P
Age (years)	1.063 (1.039 − 1.088)	<0.001	1.048 (1.014–1.083)	0.005
LVH, n (%)	3.077 (1.734 − 5.459)	<0.001	3.741 (1.828–7.657)	<0.001
hs-CRP		0.001		0.014
Low risk	REF		REF	
Intermediate risk	1.205 (0.561 − 2.589)	0.632	1.514 (0.59–3.87)	0.388
High risk	3.013 (1.477 − 6.145)	0.002	3.238 (1.349–7.768)	0.009

### hs-CRP and LVH alone or combined evaluation of CI in HD patients

**Figure 2.**
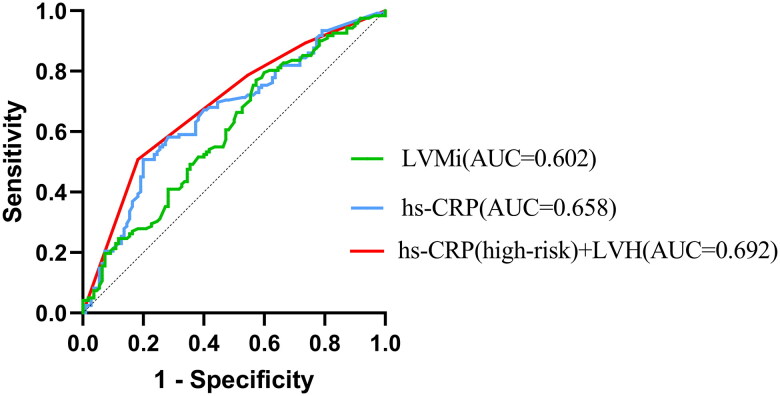
Receiver-operating characteristic (ROC) analyses.

As demonstrated in Figure 2, compared to evaluating hs-CRP or LVH alone, the combined assessment was superior to both, and yet there was no statistically significant difference.

### Synergistic effect of hs-CRP and LVH on CI in HD patients

**Figure 3.**
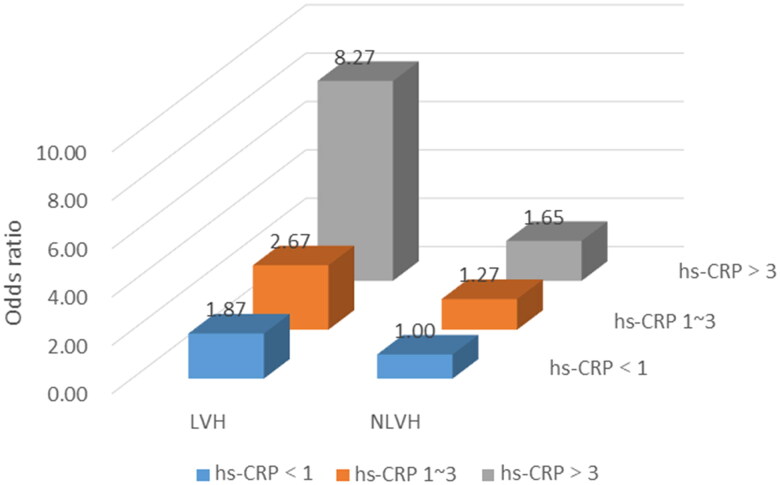
Effect of hs-CRP and ventricular classification on CI.

**Figure 4.**
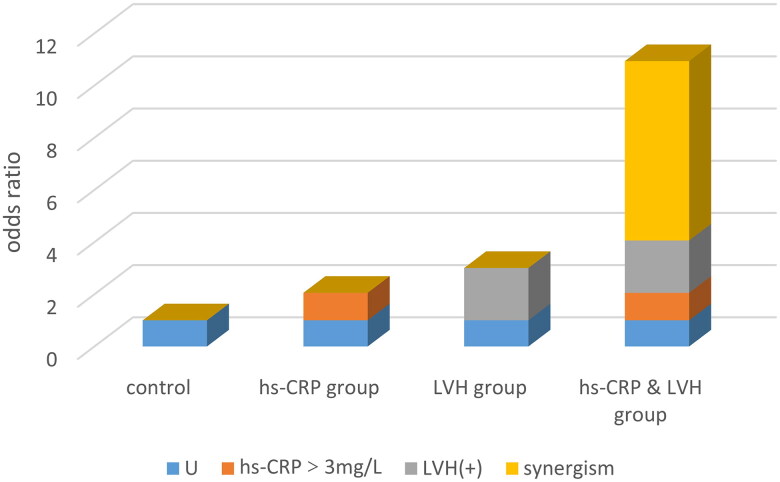
Synergistic effect of hs-CRP and LVH on CI. This figure shows the effect of different exposure categories on the risk of CI. U indicates the odds ratio (OR) of unexposed factors, as a control group, with OR = 1; the hs-CRP group indicates the CI OR of patients with hs-CRP levels >3 mg/L only. The LVH group indicates the CI OR of patients with LVH only. The hs-CRP and LVH group indicates the CI OR of patients exposed to both conditions.

Figure 3 demonstrates the predominance ratio (OR value) of different classifications of hs-CRP and ventricle on CI. Regarding hs-CRP non-high risk (≤3.0 mg/L) and no LVH, patients with high risk (>3.0 mg/L) combined with LVH had the highest prevalence of CI, which was 8.27 times higher than that of the control group. Referring to previous literature, we extended the adjustment for education by correcting for age, sex, and education. We found that for CI, there was a significant excess superimposed risk when hs-CRP levels were greater than 3 mg/L and LVH was present, with a synergy index of 6.67 (95% CI 1.05–42.31). The relative excess risk due to interaction was 6.01 (95% CI 0.01–12.01). The attributable proportion of synergy was 74% (95% CI 0.51–0.98) (Figure 4).

## Discussion

The present study reaffirms that both high CRP and LVH are important determinants of CI events, and this relationship remains evident even after adjusting for confounders by multivariate analysis. In addition, the present study demonstrated that the combination of elevated CRP and LVH has an added predictive value (synergistic effect) for CI in HD patients. These findings suggest that the coexistence of high-risk hs-CRP (>3 mg/L) and LVH is associated with a higher risk of CI than any single factor and has important implications for the early recognition of CI.

While comprehensive neuropsychological testing undeniably holds the position of the gold standard for assessing cognitive functioning, it may not possess the same allure among healthcare professionals in a clinical setting compared to screening scales [33]. The Montreal Cognitive Assessment (MoCA) emerges as a concise, self-contained screening instrument specifically designed for evaluating cognitive functioning. It encompasses crucial cognitive domains and boasts superior sensitivity and specificity compared to the Mini-Mental State Examination (MMSE) [34]. Introduced to China in 2006, the MoCA underwent translation into Chinese and was adapted to suit local circumstances, with the Beijing version (MoCA-BJ) standing as the most emblematic example. The MoCA-BJ was meticulously tailored to mirror the cognitive aptitudes of the Chinese populace more accurately, incorporating culturally pertinent content and linguistic refinements. For instance, in the Drawing Tracks test, English letters (ABCDE) are substituted with the Chinese sequence of (甲乙丙丁戊), reflecting cultural nuances and linguistic particularities [35].

Hence, in this study, the MOCA-BJ scale was employed as the yardstick to classify HD patients into those with CI and those without (Table 3). Upon comparing the two groups, we observed significant disparities in both age and dialysis duration among HD patients. However, correlation analysis revealed no significant association between these variables (Supplementary Figure 1). When patients were stratified into young, middle-aged, and elderly categories using 35 and 60 years as cutoff points, the variability between groups remained insignificant (*p* = 0.53). This outcome is actually quite understandable, as while some older patients had a longer dialysis history, the difference in dialysis duration across age groups was not pronounced due to the aging population, which has led to an increased proportion of older patients among recent admissions. Prior research has established hs-CRP, hemoglobin, and albumin as indicators of systemic inflammation and nutritional status. Decreases in hemoglobin and albumin are indicative not only of malnutrition in HD patients but also of a systemic inflammatory response [36]. Our study corroborates these findings, demonstrating a significant negative correlation between hemoglobin and albumin levels with hs-CRP (Supplementary Figure 2).

The predictive significance of circulating inflammatory markers in CI has been well-established in numerous epidemiological studies [32]. In a 2-year longitudinal study of patients with CI, Gelin Xu and colleagues observed that elevated plasma CRP levels were associated with accelerated cognitive decline and an increased risk of dementia [9]. Similarly, in a population-based study, Rosebud O. Roberts found a strong correlation between plasma CRP and CI, hypothesizing that CRP plays a role in the underlying pathology of CI [37]. Our current study also revealed a significant negative correlation between hs-CRP levels and Moca-BJ scores, further confirming hs-CRP as an independent risk factor for CI. In a recent longitudinal study among a retired population, Meike Stoldt and team further suggested that inflammation may partially mediate the relationship between education and cognition [38]. Unlike the general population, which maintains a relatively stable internal environment, HD patients experience a unique situation where their survival relies on a 4-h treatment session only 3–4 times a week. This regimen leads to numerous abrupt changes in their internal milieu, including chronic inflammation, oxidative stress, metabolic acidosis, vitamin D deficiency, anemia, and uremic toxins, among others. These factors pose significant challenges in predicting the cognitive abilities of HD patients. Hence, to mitigate the impact of inflammatory fluctuations, it is imperative to incorporate a stable indicator, thereby enhancing the precision of our predictions.

Cardiac ultrasound stands as a noninvasive and swift diagnostic tool, capable of furnishing direct evidence of the presence and extent of subclinical cardiac failure [39]. This technique holds the promise of enhancing personalized risk assessments and therapeutic interventions with greater precision. Prior research conducted by Merrill F. Elias, Simin Mahinrad, and others has consistently shown a marked correlation between an increasing LVMI and cognitive decline [16,20]. In a longitudinal study that focused on stroke-free patients, F.W. Unverzagt and colleagues observed that LVH was predictive of the development of clinically significant cognitive dysfunction. Notably, this association between LVH and cognitive impairment remained robust even after accounting for factors such as age, education, and other pertinent variables [40]. Comparable findings have been documented in patients with renal failure, and our previous study similarly revealed a strong link between LVH and CI in patients undergoing peritoneal dialysis [41]. Yoshio IWASHIMA, in a prospective cohort study, demonstrated that a combined assessment of hs-CRP and LVMI provided a superior prediction of cardiovascular and cerebrovascular risks compared to a single indicator alone, indicating that the concurrent use of these two indices yields more meaningful insights into CI [42]. Additionally, this study also uncovered a synergistic effect of these two indicators on CI risk.

There is a synergistic effect between hs-CRP and LVH in terms of CI risk. This finding suggests that hs-CRP and LVH exacerbate each other and that there is a longitudinal relationship between hs-CRP and LVH [13,43]. Multiple pathophysiologic mechanisms linking inflammation and LVH have been proposed, confirming that cardiac hypertrophy may be at least partially attributable to elevated hs-CRP levels. Inflammation can alter the morphology and function of vascular smooth muscle cells, resulting in atherosclerosis as well as stimulating endothelial dysfunction, thereby promoting the development of LVH [23,44]. Consequently, we posit that pathophysiologic changes in the vasculature create the conditions for the interaction between hs-CRP and LVH, thereby supporting the concept of vascular-ventricular coupling in HD patients [43].

LVH itself is a pro-inflammatory factor and may contribute to the pro-inflammatory state [15]. Elevated inflammatory markers may contribute to neurovascular unit damage. Inflammation and oxidative stress can disrupt white matter structure and function by interfering with neurovascular coupling, potentially leading to CI [45,46]. Wersching et al. found that higher levels of hs-CRP were associated with higher white matter hyperintensities (WMH) and less brain parenchymal volume. WMH are MRI indicators of white matter lesions. Moreover, the WMH There is a positive correlation between the volume and severity of WMH and patients’ CI, particularly subjective memory impairment [47]. Meanwhile, the greater the volume of WMH, the lower the patient’s speed of information processing and executive ability, and the worse the overall cognition [48]. Eleanor L.S. Conole et al. employed DNA methylation (DNAm), a method that can provide a more consistent reflection of inflammation exposure has indicated that chronic inflammation predominantly causes structural lesions in the brain’s white matter. These lesions mediate the association between inflammation and CI [49].

Repeated ischemia-reperfusion and circulatory stress during HD can contribute to chronically elevated levels of inflammatory markers, and elevated inflammatory markers further impair cardiac and cerebral function, which can result in LVH and cerebral hypoperfusion [50]. The presence of LVH further exacerbates cerebral ischemia and hypoxia, potentially accelerating cognitive decline [3], which may trigger a vicious cycle [44]. Brain SPECT employing 99mTc-HMPAO allows for a semi-quantitative assessment of cerebral perfusion. Cristina Sierra and colleagues employed this method to determine that cerebral blood flow ratios were markedly reduced in the striatal region of the brain among patients with LVH in comparison to those without LVH [51]. Chronic inadequate cerebral perfusion will contribute to the persistence of an ischemic and hypoxic microenvironment in the brain, which in turn induces a series of pathophysiological changes including neuroinflammation and disruption of the blood-brain barrier. Directly or indirectly, it results in damage and eventual death of brain tissue [50].

## Limitations

This study has several limitations. Firstly, being a single-center study, the small sample size may restrict its generalizability. Secondly, in this study, only hs-CRP was evaluated as an inflammatory marker. Compared to interleukin (IL)-6 and tumor necrosis factor (TNF)-α, hs-CRP is more readily available in the clinical workup and has demonstrated strong predictive value for cardiovascular risk. Additionally, a significant number of patients had neglected to disclose their renal tissue pathological during data collection, thereby omitting documentation of their underlying renal disease. Finally, since cross-sectional studies do not know the prior cognitive functioning of patients, we will further incorporate that data in this next series of studies to make our study more scientifically sound.

## Conclusions

This study reveals the synergistic role of hs-CRP and LVH in determining CI risk in HD patients. LVH has the potential to be beneficial as an early marker of structural or functional changes in the heart before the onset of apparent CI. Consequently, we propose that a combined evaluation of hs-CRP and LVH should be considered to facilitate early identification and intervention in CI. The current study holds significant implications for comprehending the pathophysiological mechanisms linking chronic inflammation with LVH and CI.

## Supplementary Material

Figure4.tif

Supplementary Table.docx

Supplementary Figure1.tif

Figure2.tif

Figure1.tif

Figure3.tif

Supplementary Figure2.tif

Visual Abstract.tif

## Data Availability

All data generated or analyzed during this study are included in this article and its supplementary information files. Further enquiries can be directed to the corresponding author.

## Declarations

Ethics approval and consent to participate
