# Supplementary figures and images for: Effects of high-sensitivity C-reactive protein and left ventricular hypertrophy on cognitive function in hemodialysis patients
Source: Ren Fail. 2025 Jan 16;47(1):2450522. doi: 10.1080/0886022X.2025.2450522 (PMC11749012; doi:10.1080/0886022X.2025.2450522)

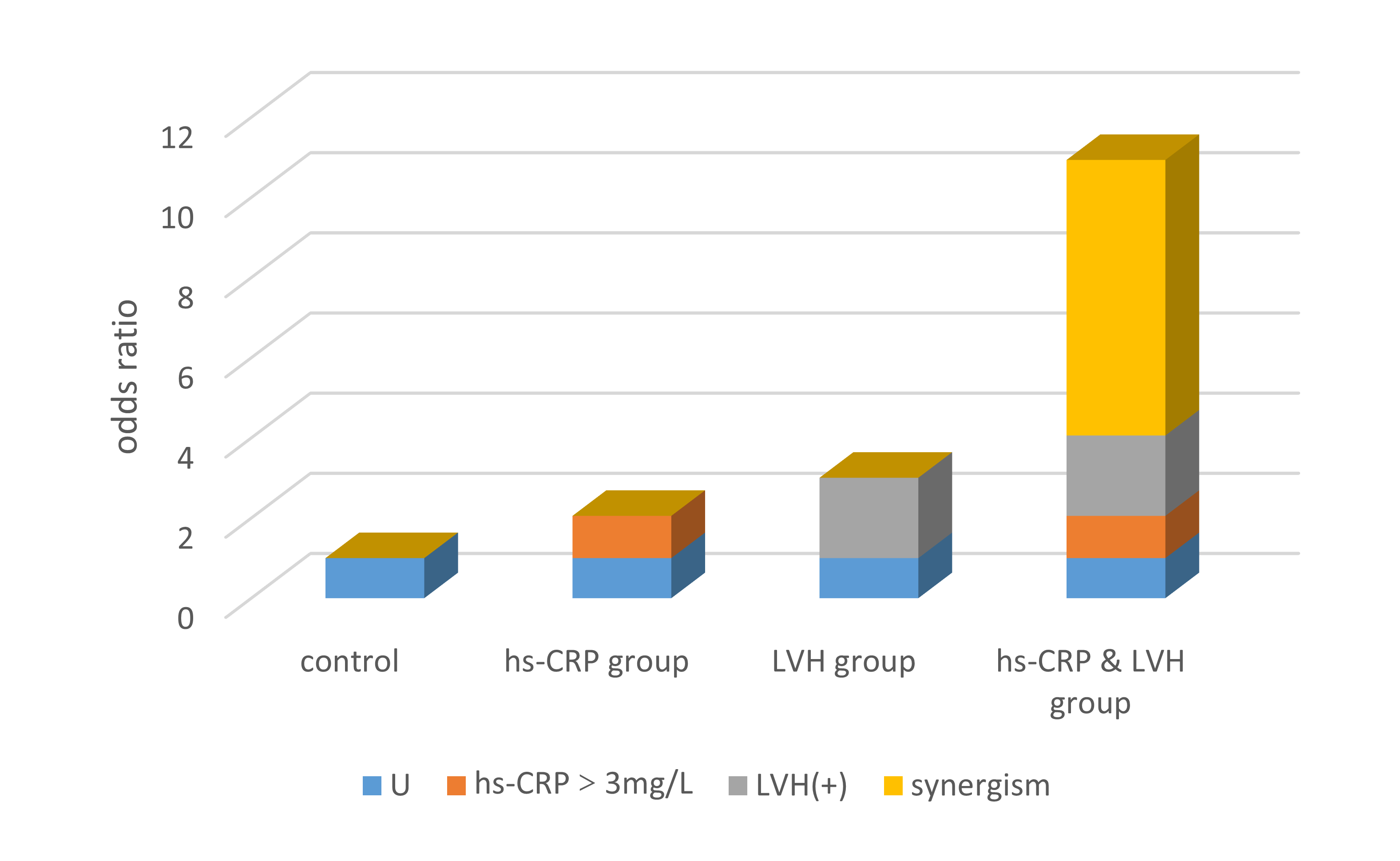

Supplement: Figure4.tif [file IRNF_A_2450522_SM1592.tif]

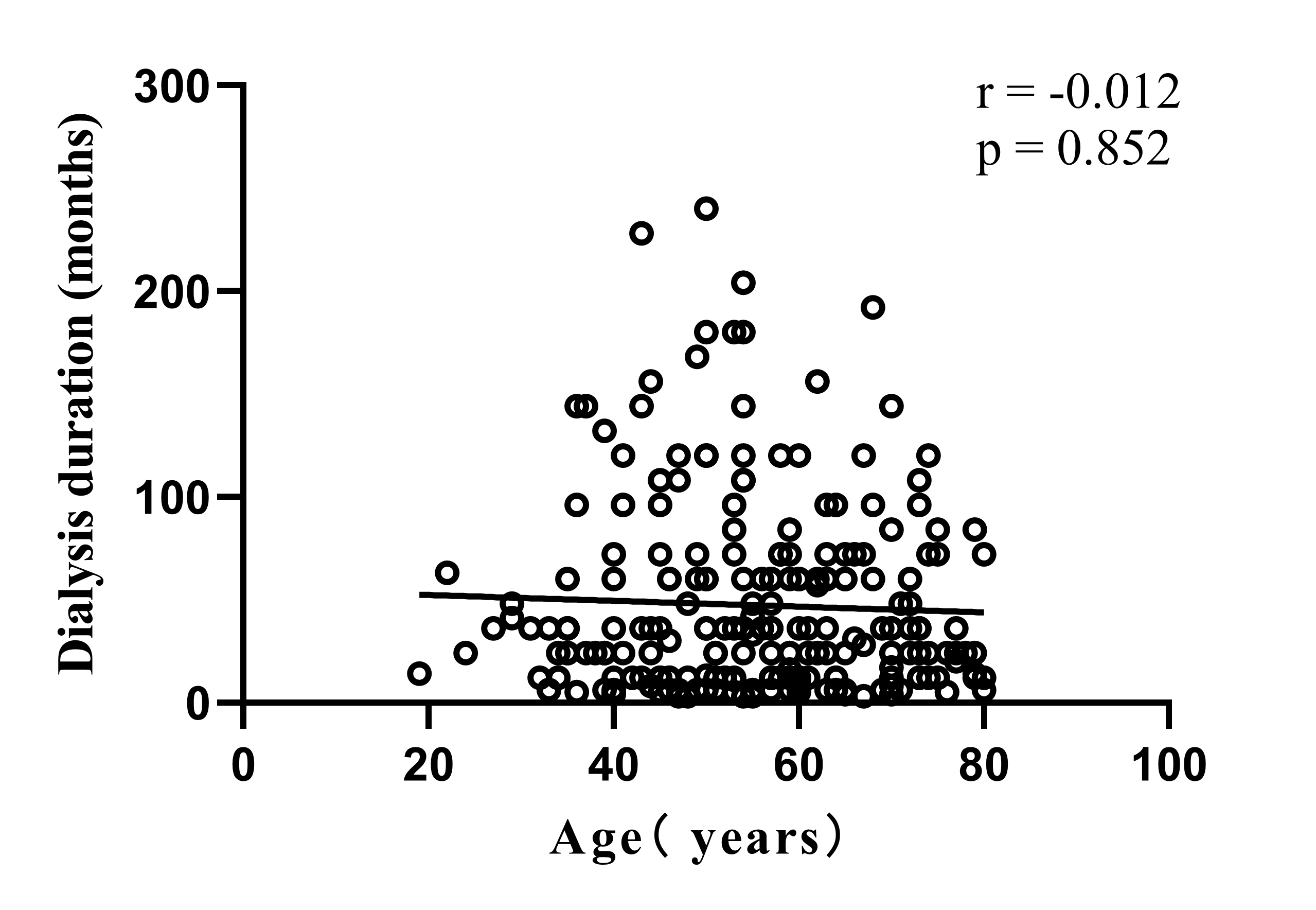

Supplement: Supplementary Figure1.tif [file IRNF_A_2450522_SM1590.tif]

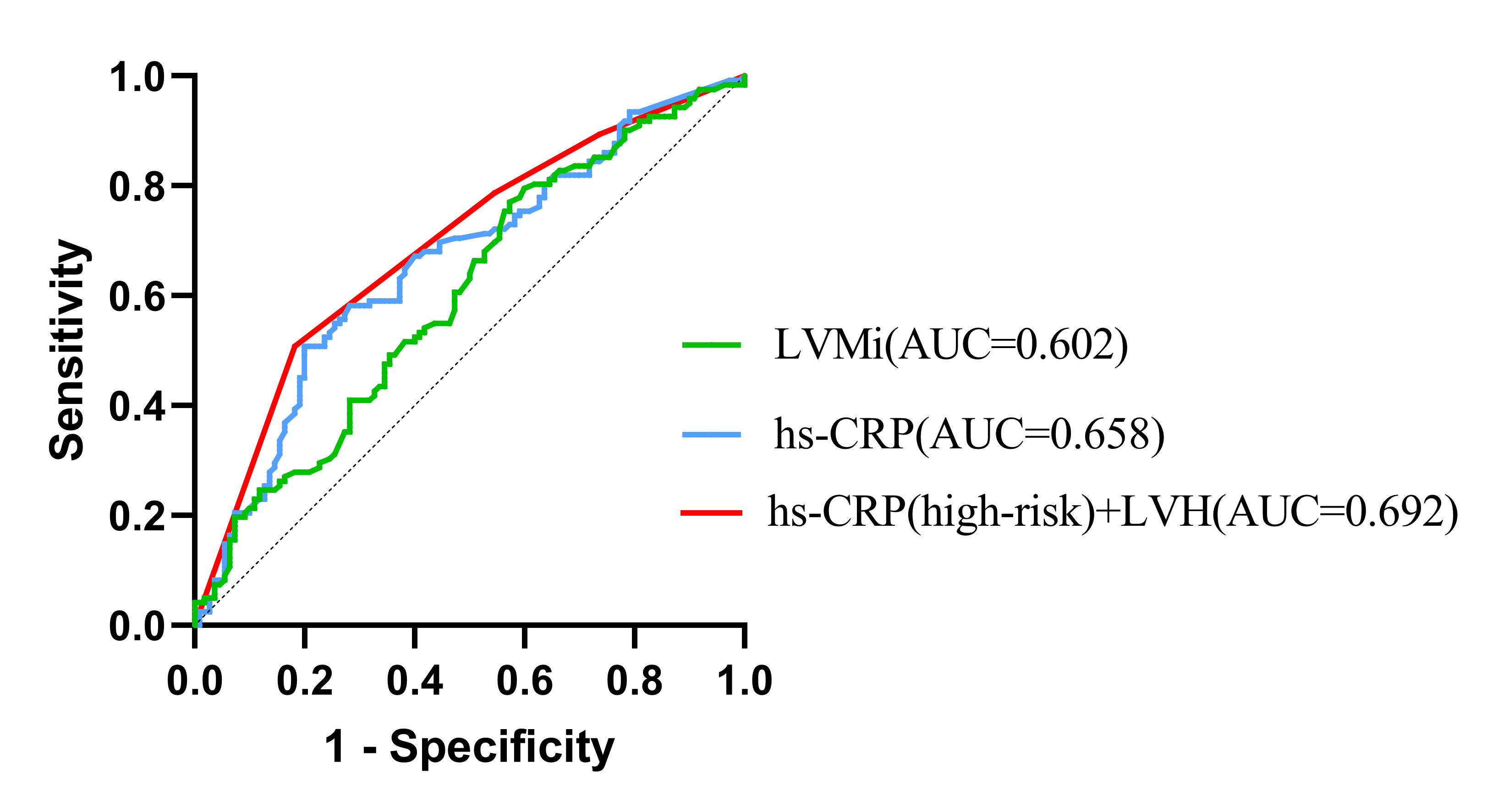

Supplement: Figure2.tif [file IRNF_A_2450522_SM1589.tif]

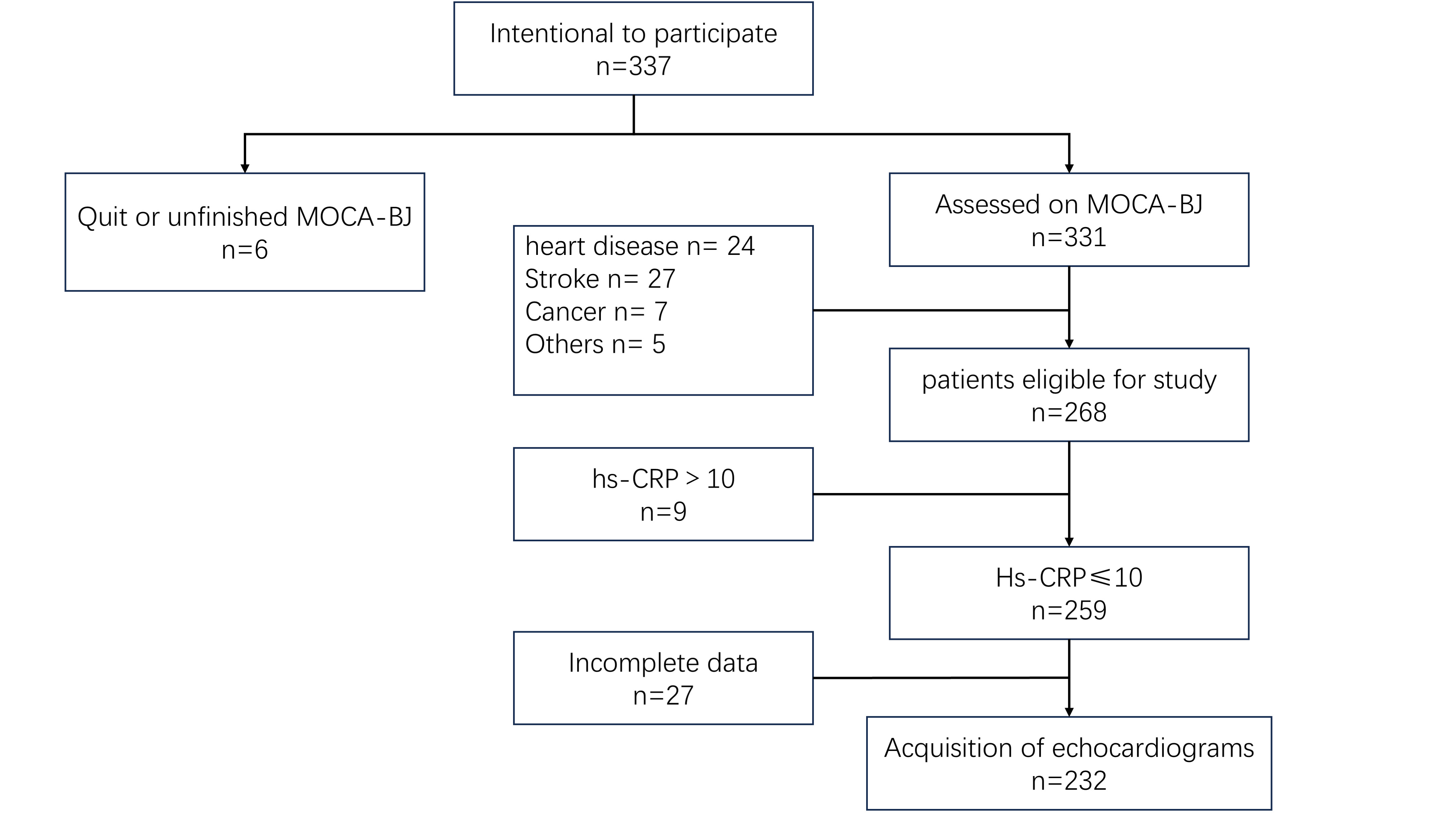

Supplement: Figure1.tif [file IRNF_A_2450522_SM1588.tif]

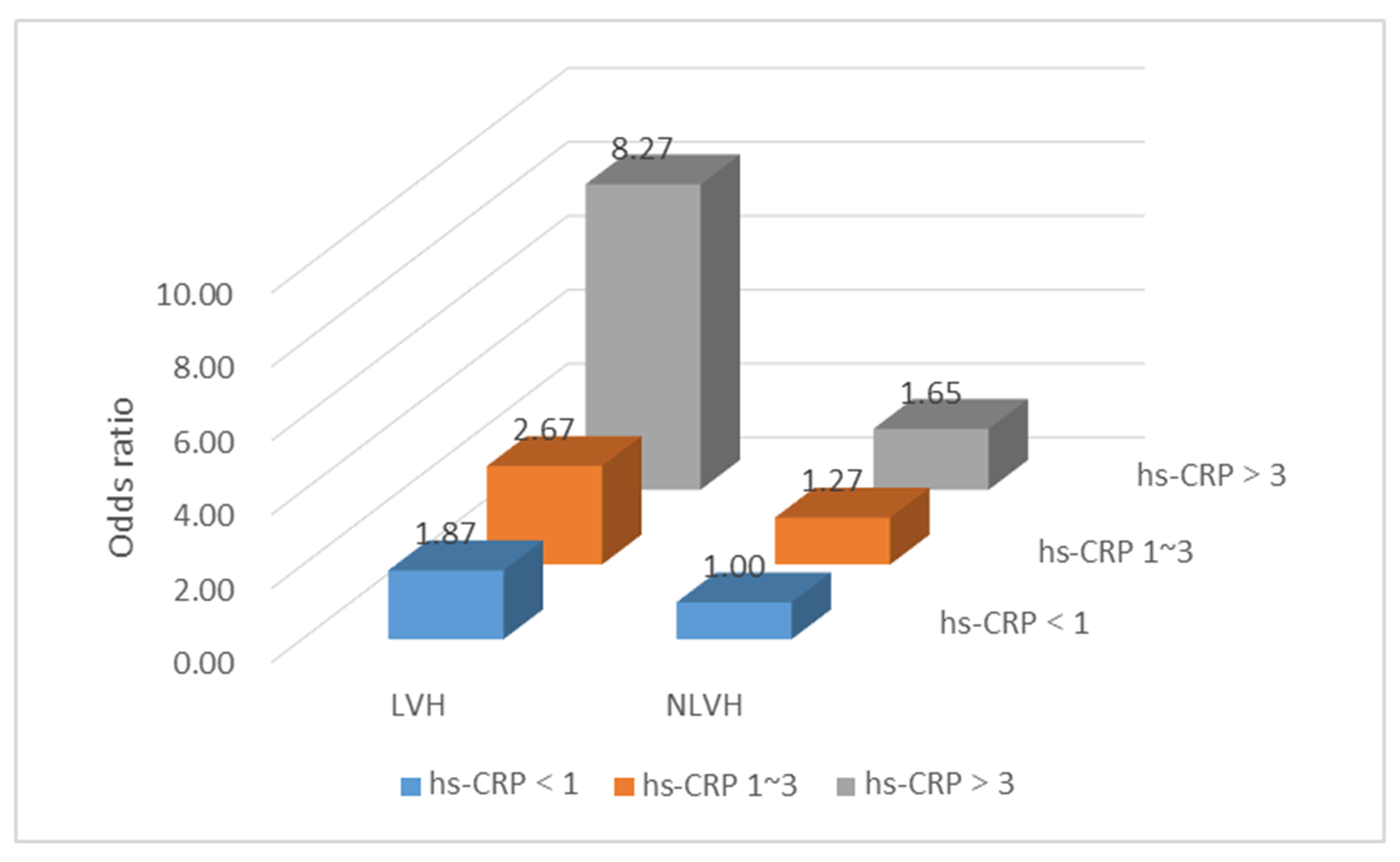

Supplement: Figure3.tif [file IRNF_A_2450522_SM1587.tif]

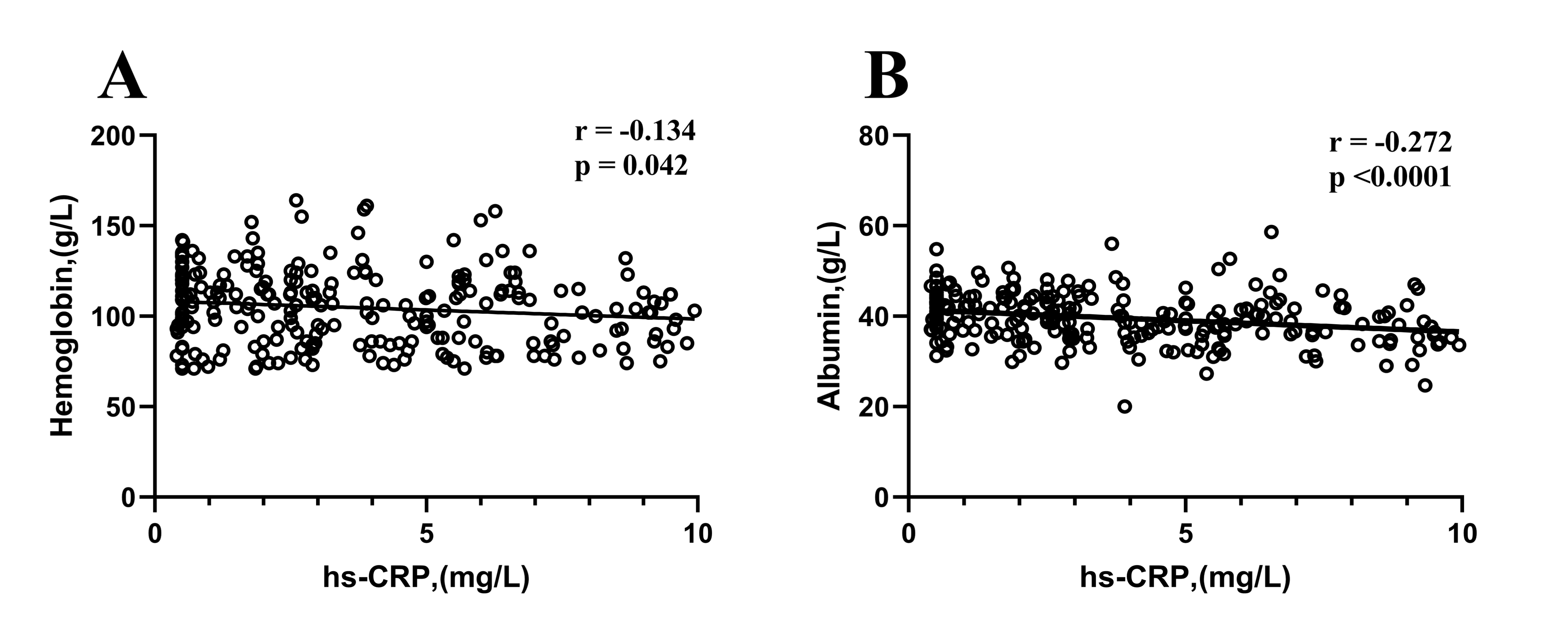

Supplement: Supplementary Figure2.tif [file IRNF_A_2450522_SM1586.tif]

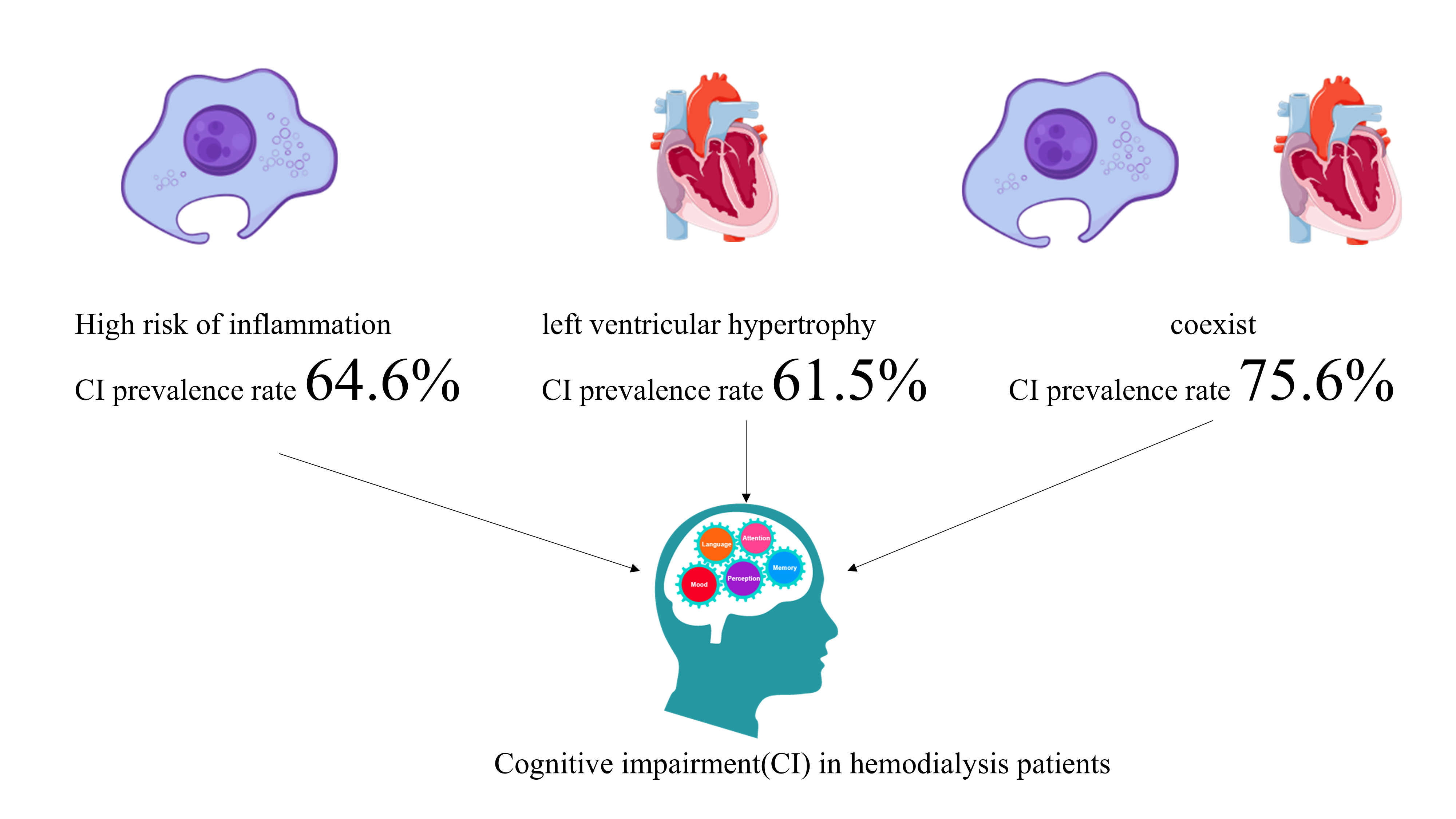

Supplement: Visual Abstract.tif [file IRNF_A_2450522_SM1585.tif]
