# Supplementary material for: Effects of high-sensitivity C-reactive protein and left ventricular hypertrophy on cognitive function in hemodialysis patients
Source: Ren Fail. 2025 Jan 16;47(1):2450522. doi: 10.1080/0886022X.2025.2450522 (PMC11749012; doi:10.1080/0886022X.2025.2450522)
Supplement: Supplementary Table.docx [file IRNF_A_2450522_SM1591.docx]

Supplementary Table 1: Logistic regression on the determinant factors of CI

|  | Univariate analysis | | Multivariate analysis | |
| --- | --- | --- | --- | --- |
|  | OR (95% CI) | P | OR (95% CI) | P |
| Age, (years) | 1.063 (1.039 - 1.088) | ＜0.001 | 1.048 (1.014 - 1.083) | 0.005 |
| Males, n (%) | 0.939 (0.559 - 1.58) | 0.814 | 1.309 (0.678 - 2.527) | 0.423 |
| Education level,  n (%) |  | ＜0.001 |  | 0.233 |
| ≤Elementary school | REF |  | REF |  |
| Middle school | 0.288 (0.146 - 0.569) | ＜0.001 | 0.434 (0.189 - 1.0) | 0.05 |
| High school | 0.231 (0.102 - 0.521) | ＜0.001 | 0.42 (0.15 - 1.173) | 0.098 |
| ＞High school | 0.178 (0.064 - 0.492) | ＜0.001 | 0.505 (0.143 - 1.785) | 0.289 |
| BMI, (kg/m2) | 1.009 (0.941 - 1.083) | 0.801 | 1.042 (0.957 - 1.135) | 0.343 |
| LVH, n (%) | 3.077 (1.734 - 5.459) | ＜0.001 | 3.741 (1.828 - 7.657) | ＜0.001 |
| Dialysis duration, (months) | 1.006 (1.0 - 1.011) | 0.063 | 1.007 (1.0 - 1.014) | 0.058 |
| ACEI/ARB inhibitors, n (%) | 0.532 (0.274 - 1.032) | 0.062 | 0.763 (0.344 - 1.69) | 0.505 |
| hs-CRP |  | 0.001 |  | 0.014 |
| Low risk |  | REF |  | REF |
| Moderate risk | 1.205 (0.561 - 2.589) | 0.632 | 1.514 (0.59 - 3.87) | 0.388 |
| High risk | 3.013 (1.477 - 6.145) | 0.002 | 3.238 (1.349 - 7.768) | 0.009 |
| Albumin, (g/L) | 0.94 (0.895 - 0.988) | 0.015 | 1.002 (0.936 - 1.071) | 0.964 |
| Potassium, (mmol/L) | 0.616 (0.421 - 0.902) | 0.013 | 0.758 (0.473 - 1.213) | 0.248 |
| Glycated hemoglobin, (%) | 0.984 (0.972 - 0.997) | 0.016 | 0.989 (0.973 - 1.006) | 0.213 |
| HR, (beats/min) | 0.982 (0.962 - 1.002) | 0.079 | 0.985 (0.96 - 1.012) | 0.274 |
| DBP, (mmHg) | 0.974 (0.954 - 0.995) | 0.017 | 0.985 (0.958 - 1.012) | 0.27 |
| Diastolic dysfunction, n (%) | 2.269 (1.112 - 4.63) | 0.024 | 1.235 (0.485 - 3.145) | 0.658 |

BMI Body mass index; LVH Left ventricular hypertrophy; ACEI Angiotensin Converting Enzyme Inhibitor; ARB Angiotensin Receptor Blocker; HR Heart rate; DBP Diastolic Blood pressure
